# Schizophrenia in Malaysian families: A study on factors associated with quality of life of primary family caregivers

**DOI:** 10.1186/1752-4458-5-16

**Published:** 2011-06-08

**Authors:** Ruzanna ZamZam, Marhani Midin, Lim S Hooi, Eng J Yi, Siti NA Ahmad, Siti FA Azman, Muhammad S Borhanudin, Rozhan SM Radzi

**Affiliations:** 1 Department of Psychiatry, Faculty of Medicine, Universiti Kebangsaan Malaysia, Kuala Lumpur 56000, Malaysia; 2 Department of Community Health, Faculty of Medicine, Universiti Kebangsaan Malaysia, Kuala Lumpur 56000, Malaysia

## Abstract

**Background:**

Schizophrenia is a chronic illness which brings detrimental effects in the caregivers' health. This study was aimed at highlighting the socio-demographic, clinical and psychosocial factors associated with the subjective Quality of Life (QOL) of Malaysian of primary family caregivers of subjects with schizophrenia attending an urban tertiary care outpatient clinic in Malaysia.

**Methods:**

A cross-sectional study was performed to study patient, caregiver and illness factors associated with the QOL among 117 individuals involved with caregiving for schizophrenia patients. The study used WHOQOL-BREF to assess caregivers' QOL and Brief Psychiatric Rating Scale (BPRS) to assess the severity of patients' symptoms. Social Readjustment Rating Scale (SRRS) assessed the stress level due to life events.

**Results:**

The mean scores of WHOQOL-BREF in physical, psychological, social and environmental domains were 66.62 (14.36), 61.32 (15.52), 62.77 (17.33), 64.02 (14.86) consecutively. From multiple regression analysis, factors found to be significantly associated with higher QOL were higher educational level among caregivers in social and environmental domains; caregivers not having medical problem/s in physical and psychological domains; later onset and longer illness duration of illness in social domains; patients not attending day care program in environmental domain; lower BPRS score in physical and environmental domains. SRRS score of caregivers was also found to have a significant negative correlation with QOL in environmental and psychological domains. Other factors were not significantly associated with QOL.

**Conclusion:**

Caregivers with more social advantages such as higher educational level and physically healthier and dealing with less severe illness had significantly higher QOL in various aspects. Supporting the caregivers in some of these modifiable factors in clinical practice is important to achieve their higher level QOL.

## Introduction

Schizophrenia is a chronic and profoundly disabling psychiatric disorder (1). In Malaysia, there are increasing number of people with newly diagnosed schizophrenia receiving psychiatric care (2, 3). Most of schizophrenia patients have impairment in social functioning and this is known to cause distress not only to the patients themselves but also to the caregivers (4-7).

Previous studies concluded that caregivers of schizophrenia patients were at risk of having lower QOL due to mental health problems and higher caregiver burden (8, 9). Their subjective QOL was found to be similar to that of the patients but lower than that of a general population sample. (10)

The level of QOL in caregivers of the mentally ills has been found to be associated with various factors including illness factors in patients and psychosocial background of the caregivers. Severity of symptoms, illness duration, level of disabilities, perceived stigma (11-18), being female caregivers with nuclear family (19), being older with lower socioeconomic status (12) and recent life crisis (20) were found to be associated with lower QOL.

The issue of QOL in caregivers is particularly important in Malaysia as similar to what is happening in many other neighboring countries, the mental health care system in Malaysia is now moving to the community-based care where families take up bigger role in caring for patients and may experience additional burden(21). This process of change, which has been guided by a clear policy (22) and legal act (23) requires families to actively participate in planning and management of patients (24). Even though skill training, psychoeducation and emotional support in handling the mentally ill patients at home are being given to caregivers, these may not be enough to equip them with the new challenge of having patients constantly at home. Admittedly, there is still shortage of community resources to cater for the needs of caregivers and caregiving still requires much time, effort and work on the care givers part (25). Another worrying situation is even when Malaysian caregivers developed significant distress, they do not complaint about it (26) and this may lead to burn out in caregiving and other negative consequences.

In this context of change in service system where families are taking up a bigger role in caregiving of patients, this study was designed to assess the various factors that affect the QOL of Malaysian caregivers while caring for family member with schizophrenia. It can be informative to the service providers in implementing more effective family interventions.

## Methods

This was a cross-sectional study, conducted at psychiatry outpatient clinic in Universiti Kebangsaan Malaysia Medical Center (UKKMC), Kuala Lumpur, from November 2009 till April 2010. This is an urban tertiary facility, located south of Kuala Lumpur, a government run center which is easily accessible by public transports. It provides comprehensive medical care including psychiatry.

## Sample

All consecutive patients with schizophrenia and their primary family caregivers who came for clinic visits during the study period were offered to enter the study. Primary family caregiver was defined as the person belonging to the patient's family system who took the care and was responsible for the patient, and who committed most of his or her time to that task without receiving any economic retribution (27). They were identified by researchers using the clinic patient registry. Both the caregivers and patients who fulfilled the inclusion criteria were explained about the study and consents were obtained. The inclusion criteria were: patients who diagnosed with schizophrenia based on Diagnostic Statistical Manual of Mental Disorder-IV-TR (DSM-IV-TR) by psychiatrist; clinically stable to provide consent and to participate in this study. The exclusion criteria were those who declined consent and not accompanied by their primary family caregiver.

## Instruments

Quality of life questionnaire (WHOQOL-BREF) measured the main study outcome. It is a shortened version of WHOQOL-100, developed by WHOQOL group. There are four main domains derived from the 26 items in this questionnaire, comprising of physical, psychological, social and environment. These four domains were shown to be valid measures of overall QOL and health. This questionnaire is cross-culturally sensitive has good to excellent reliability and validity (28). There are 19 different languages available and are self-administered. Higher score means a better quality of life. In this study, the Malay version of WHOQOL_BREF was used. It has been validated and showed high correlation with that of WHOQOL-100. It was found to have good discriminant and construct validity, internal consistency and test-retest reliability (29).

Brief Psychiatric Rating Scale (BPRS) evaluated the patients' psychopathology. The self-rated Social Readjustment Rating Scale (SRRS)(30). It is the most widely used instrument for the measurement of an individual's experience of psychosocial stress. In our local context, SRRS was used in local study and there was a remarkable concordance (Spearman's rho ranged from. 97 to. 91) between the Malaysian and American samples (31). We used the modified Malay version of SRRS (32) in this study, which has been widely used in local studies (33, 34).

## Procedure

Both patients and their caregiver completed the WHOQOL-BREF Malay version and SRRS Malay version. Five investigators conducted structured interviews with the patients to assess the severity of their conditions using BPRS. The interviews were conducted in Malay language as all subjects and caregivers could speak Malay regardless of their ethnicity. This research project was approved by the Research and Ethical Committee, Faculty of Medicine, PPUKM.

## Statistical analysis

Data entry and statistical analysis were conducted using Statistical Package for Social Sciences (SPSS) program for windows, version 18.0. The distribution of each domain of WHOQOL-BREF was described. Bivariate analysis was performed for each independent factor using student t-test analyses. All the significant factors later further analyzed using linear regression analysis. The inclusion of variables in the model was based on p value less than 0.05.

## Results

Out of a total of 209 patients approached, 92 patients who fulfilled the exclusion criteria were excluded. Amongst them, 50 came alone without caregivers, 29 did not give their consent, whilst the rest 13 were not the primary caregivers. A total o 117 patients with their caregivers who met the inclusion criteria, were enrolled for this study.

Slightly more than half of the patients and caregivers were females (Table 1). On average, the caregivers were in their middle age and most of the patients were young adults. In terms of ethnic distribution, Malays and Chinese were predominant. This represents the ethnic distribution of the local population in the study area. The majority of patients was single, unemployed, not receiving financial aid and had received education up to the secondary school level. Most of the caregivers were parents, married, did not have medical problems and half of them were employed. Almost 90% of caregivers were also providing care to two and more either financially or socially dependent family members.

**Table 1:** Sociodemographic and clinical profiles of the respondents

	Patients		Caregivers	
	N	%	N	%
**Demographic variable**				
Gender				
Male	49	41.9	56	47.9
Female	68	58.1	61	52.1
				
Age				
≤ 45 years	77	65.8	34	29.1
> 45 years	40	34.2	83	70.9
				
Race				
Malay	49	41.9	49	41.9
Others	68	58.1	68	58.1
				
Religion				
Islam	52	44.4	52	44.4
Others	65	55.6	14	7.7
				
Marital status				
Married	30	25.6	80	68.4
Single/Divorced/Widowed	87	74.4	37	31.6
				
Educational level				
Lower (Non/Primary)	33	28.2	32	27.4
Higher (Secondary/Tertiary)	84	71.8	85	72.6
				
Employment status				
Yes	23	19.7	60	51.3
No	94	80.3	57	48.7
				
Financial aid				
Yes	11	9.4	-	-
No	106	90.6	-	-
				
Relationship with patients				
Parents	-	-	51	43.6
Others	-	-	66	56.4
				

**Illness variable**				
Duration of illness				
<10 years	53	45.3	-	-
≥ 10 years	64	54.7	-	-
				
Onset of illness				
< 45	102	87.2	-	-
> 45	15	12.8	-	-
				
No of hospitalization				
< 4	105	89.7	-	-
≥ 4	12	10.3	-	-
				
Attending day care				
Yes	16	13.7	-	-
No	101	86.3	-	-
				
Types of medication				
Typical/Mixed	37	31.6	-	-
Atypical	80	68.4	-	-
				

	Mean score	SD	Mean score	SD

WHOQOL total	-	-	254.74	52.50
Physical	-	-	66.62	14.36
Psychological	-	-	61.32	15.52
Social	-	-	62.72	17.33
Environmental	-	-	64.02	14.86
BPRS	27.82	8.24		
SRRS	107.66	86.92		
				

N = number of respondents

SD = standard deviation

About half of the patients had had the illness for ten years or more. Only 10% of the patients had had four or more psychiatric hospitalizations in the past. About 14% attended one or the other rehabilitation activities at the hospital day care. A majority of them (60%) were receiving atypical antipsychotics.

In terms of the QOL scores, the means scores (standard deviation (SD)) of physical, psychological, social and environmental domains were 66.62 (14.36), 61.32 (15.52), 62.77 (17.33), 64.02 (14.86) consecutively. The mean scores (SD) of BPRS and SRRS were 27.82 (8.24) 107.66 (86.92) respectively. From bivariate analyses, a number of sociodemographic and clinical factors were found to be significantly associated with one or more domains of WHOQOL-BREF (Table 2). Duration of illness of less than ten years and patients not attending day care program were significantly associated with higher scores in all domains of the caregivers' QOL. Caregivers who received higher education (secondary level or higher) significantly had higher QOL scores in all domains. Caregivers who did not have medical problems significantly had higher QOL scores in physical, psychological and environmental. BPRS scores in patients were significantly and inversely correlated with physical and environmental domains of QOL (table 3) and SRRS scores in caregivers showed similar relationship with psychological and environmental domains.

**Table 2:** Patient, Caregiver and illness factors and caregivers' Quality of Life

	Physical domain			Psychological domain			Social domain			Environmental domain		
	**Mean ± SD**	**t**	***p*^a^**	**Mean ± SD**	**t**	***p*^a^**	**Mean ± SD**	**t**	***p*^a^**	**Mean ± SD**	**t**	***p*^a^**

**Patient factor**												
Age												
≤ 45 years (77) > 45 years (40)	65.90 ± 14.96 65.03 ± 13.22	-0.789	0.432	61.53 ± 16.23 60.93 ± 13.08	0.216	0.829	62.35 ± 17.46 63.58 ± 17.27	-0.362	0.718	63.49 ± 14.98 65.03 ± 14.76	-0.530	0.598
Gender												
Male (49) Female (68)	64.82 ± 15.63 67.93 ± 13.34	-1.128	0.262	61.02 ± 15.53 61.54 ± 15.63	-0.719	0.858	61.61 ± 14.80 63.60 ± 19.02	-0.611	0.542	63.53 ± 15.43 64.37 ± 14.54	-0.297	0.767
Marital status												
Married (30) Single/Divorced/Widowed (87)	68.50 ± 11.47 65.98 ± 15.24	0.950	0.346	64.37 ± 13.60 60.28 ± 16.07	1.354	0.181	59.33 ± 17.69 63.95 ± 17.15	-1.243	0.220	64.57 ± 14.31 63.83 ± 15.42	0.252	0.802
Employment status												
Yes (23) No (94)	69.43 ± 16.34 65.94 ± 13.85	0.947	0.351	68.96 ± 15.53 59.46 ± 15.02	2.646	0.012*	62.78 ± 15.50 62.77 ± 17.83	0.004	0.996	68.57 ± 15.47 62.90 ± 14.57	1.591	0.121
Financial aid												
Yes (11) No (106)	65.55 ± 15.08 66.74 ± 14.36	-0.250	0.807	61.09 ± 15.16 61.35 ± 15.63	-0.054	0.958	59.00 ± 14.12 63.16 ± 17.64	-0.906	0.381	63.82 ± 19.69 64.04 ± 14.39	-0.036	0.972
Educational level												
Primary (33) Secondary and above (84)	63.21 ± 14.64 67.96 ± 14.12	-1.596	0.116	55.58 ± 13.54 63.58 ± 15.74	-2.746	0.008*	57.55 ± 18.85 64.52 ± 16.37	-1.948	0.057	60.18 ± 16.35 65.52 ± 14.04	-1.652	0.105
**Caregiver factor**												
Age												
≤ 45 years (34) > 45 years (83)	68.41 ± 13.12 65.89 ± 14.86	0.907	0.368	62.03 ± 15.29 61.04 ± 15.70	0.317	0.753	61.91 ± 15.91 63.12 ± 17.96	-0.359	0.721	62.44 ± 13.22 64.65 ± 15.57	-0.769	0.444
Gender												
Male (56) Female (61)	69.46 ± 13.61 64.02 ± 14.65	2.085	0.039*	62.71 ± 13.91 60.05 ± 16.88	0.935	0.352	64.16 ± 16.20 61.49 ± 18.35	0.835	0.405	64.29 ± 13.77 63.77 ± 15.91	0.188	0.851
Marital status												
Married (80) Single/Divorced/Widowed (37)	67.63 ± 13.70 64.46 ± 15.68	1.055	0.295	62.61 ± 16.12 58.54 ± 13.94	1.397	0.166	64.00 ± 19.06 60.11 ± 12.67	1.306	0.194	65.51 ± 15.33 60.78 ± 13.41	1.693	0.094
Educational level												
Primary (32) Secondary and above (85)	59.41 ± 12.64 69.34 ± 14.10	-3.670	0.001*	55.00 ± 13.30 63.71 ± 15.70	-3.000	0.004*	55.66 ± 16.32 65.45 ± 17.03	-2.858	0.006*	56.25 ± 13.91 66.94 ± 14.21	-3.685	0.001*
Employment status												
Yes (60) No (57)	70.42 ± 14.10 62.63 ± 13.66	3.034	0.003*	64.18 ± 14.84 58.32 ± 15.78	2.069	0.041*	63.02 ± 16.03 62.51 ± 18.75	0.157	0.875	64.92 ± 14.22 63.07 ± 15.57	0.669	0.505
Medical problems												
Yes (33) No (84)	57.24 ± 15.48 70.31 ± 12.13	-4.351	0.000*	53.73 ± 15.44 64.31 ± 14.59	-3.388	0.001*	58.88 ± 16.54 64.30 ± 17.49	-1.568	0.122	58.85 ± 15.30 66.05 ± 14.26	-2.333	0.023*
Dependent members												
< 2 (12) ≥ 2 (105)	65.25 ± 14.52 66.78 ± 14.41	-0.346	0.728	62.00 ± 12.02 61.25 ± 15.92	0.198	0.846	68.83 ± 13.35 62.08 ± 17.65	1.601	0.129	64.08 ± 15.37 64.01 ± 14.88	0.016	0.988
Relationship with patients												
Parents (51) Others (66)	62.45 ± 15.23 69.85 ± 12.87	-2.785	0.006*	58.22 ± 16.38 63.73 ± 14.49	-1.897	0.061	62.25 ± 18.63 63.17 ± 16.40	-0.276	0.783	62.24 ± 15.83 63.39 ± 14.03	-1.124	0.264

		**r**	***p*^b^**		**r**	***p*^b^**		**r**	***p*^b^**		**r**	***p*^b^**

SRRS		-0.1660	0.086		-0.205	0.027*		-0.146	0.115		-0.233	0.012*
												
**Illness factor**												
No of hospitalisation												
< 4 (105) ≥ 4 (12)	66.98 ± 14.19 63.50 ± 16.13	0.717	0.486	61.96 ± 15.11 55.75 ± 18.52	1.120	0.283	64.06 ± 17.17 51.50 ± 15.07	2.694	0.017*	65.13 ± 14.44 54.25 ± 15.47	2.333	0.037*
Duration of illness												
< 10 years (53) ≥ 10 years (64)	70.08 ± 12.67 63.77 ± 15.14	2.455	0.016*	64.91 ± 16.05 58.36 ± 14.53	2.291	0.024*	68.87 ± 15.65 57.72 ± 17.14	3.673	0.000*	67.47 ± 14.63 61.16 ± 14.54	2.331	0.022*
Onset of illness												
< 45 years (102) > 45 years (15)	65.73 ± 14.74 72.73 ± 9.84	-2.392	0.025*	60.96 ± 16.08 63.80 ± 11.09	-0.866	0.395	61.27 ± 16.87 72.93 ± 17.60	-2.409	0.027*	63.13 ± 14.62 70.07 ± 15.58	-1.623	0.122
Attending day care												
Yes (16) No (101)	57.94 ± 15.49 68.00 ± 13.76	-2.450	0.024*	53.19 ± 14.78 62.61 ± 15.31	-2.359	0.028*	54.69 ± 14.04 64.05 ± 17.52	-2.389	0.025*	52.50 ± 14.54 65.84 ± 14.14	-3.424	0.003*
Types of medication												
Typical/mixed (37) Atypical (80)	65.95 ± 16.22 66.94 ± 13.52	-0.324	0.747	59.14 ± 13.00 62.34 ± 16.54	-1.133	0.260	59.65 ± 15.62 64.21 ± 17.98	-1.400	0.166	61.73 ± 13.22 65.08 ± 15.51	-1.201	0.233

		**r**	***p*^b^**		**r**	***p*^b^**		**r**	***p*^b^**		**r**	***p*^b^**

BPRS		-0.229	0.013*		-0.168	0.070		-0.181	0.051		-0.200	0.031*

SD = standard deviation

*p *= *p *value

r = Pearson correlation coefficient

*p*^a ^= from *t *test if not otherwise specified

*p*^b ^= from correlation test

**Table 3:** Correlations between WHOQOL domains and BPRS/ SSRS

	Physical domain		Psychological domain		Social domain		Environmental domain	
	
	r	p	r	p	r	p	r	p
BPRS	-0.229	0.013*	-0.168	0.070	-0.181	0.051	-0.200	0.031*
SRRS	-0.1660	0.086	-0.205	0.027*	-0.146	0.115	-0.233	0.012*

Table 4 presents the statistically significant (p < 0.05) results of the multivariate logistic regression analysis of the association between the various factors and each domain of QOL. Some of the factors which initially showed significant associations with QOL through bivariate analysis became insignificant factors. Factors that were found to be predictive of higher QOL scores in physical domain were lower BPRS scores in patient and caregivers not having medical problems. Caregivers without medical problems were again found to be a predictor of the higher QOL score in psychological domain besides higher education level among patients.

**Table 4:** Multiple regression analysis of patient, caregiver and illness factors in relation to QOL

	Physical domain			Psychological domain			Social domain			Environmental domain		
	**B**	**p**	**95% CI**	**B**	**p**	**95% CI**	**B**	**p**	**A**	**B**	**p**	**95% CI**

Attending day care	0.150	0.073	-0.58 - 13.032	0.129	0.146	-2.064 - 13.682	0.103	0.243	-3.571 - 13.942	0.228	0.009*	2.519 - 17.102
Duration of illness	-0.132	0.136	-8.782 - 1.213	-0.124	0.190	-9.626 - 1.933	-0.207	0.029*	-13.614--0.759	-0.090	0.322	-8.039- 2.666
Carer educational level	0.158	0.067	-0.359- 10.488	0.139	0.131	-1.456 - 11.087	0.203	0.028*	0.887 - 14.839	0.269	0.003*	3.110 - 14.728
Caregivers having medical problem	0.286	0.001*	3.774 - 14.389	0.197	0.031*	0.637 - 12.913	0.064	0.477	-4.369 - 9.284	0.105	0.232	-2.239 - 9.131
Onset of illness	0.075	0.396	-4.274--10.706	-0.007	0.939	-8.997 - 8.327	0.195	0.040*	0.451 - 19.719	0.079	0.389	-4.526- 11.519
Employment status	-0.116	0.221	-8.664 - 2.024	-0.056	0.579	-7.916 - 4.444	0.044	0.660	-5.344 - 8.403	0.042	0.668	-4.482 - 6.965
Being parents	0.054	0.575	-3.919 - 7.026	0.059	0.564	-4.481 - 8.175	-0.113	0.268	-10.989- 3.089	-0.046	0.642	-7.238- 4.485
Patient's educational level	0.137	0.107	-0.958 - 9.684	0.204	0.026*	0.864- 3.172	0.182	0.046*	0.129-13.818	0.150	0.090	-0.775- 10.624
No of hospitalisation	0.059	0.485	-5.084 - 10.640	-0.007	0.937	-9.457 - 8.727	-0.081	0.368	-14.722 - 5.503	-0.084	0.336	-12.522 - 4.320
BPRS	-0.204	0.017*	-0.648- -0.064	-0.512	0.096	-0.624 - 0.052	-0.517	0.085	-0.705 - 0.046	-0.167	0.059	-0.614 - 0.012
SRRS	-0.104	0.211	-0.044 - 0.010	-0.612	0.071	-0.060 - 0.002	-0.103	0.249	-0.055 - 0.014	-0.182	0.037*	-0.060 - -0.002

For social domain, duration of illness of less than ten years and later onset of illness as well as higher patient education level were predictive of higher scores. Higher scores in environmental domain was predicted by patient not attending day care program, higher educational level and higher SRRS scores among caregivers.

## Discussion

This study yielded two main findings. Firstly, among the patient and illness factors studied, shorter duration and later onset of illness, not attending day care program, lower BPRS scores and higher education among patients were found to be significant predictors of higher scores in one or more QOL domains in caregivers. Secondly, caregivers with higher educational level, not having medical problems and facing less social readjustment to recent life events were predictive of higher QOL in one or more domains.

Course of the illness, lack of social support and recent life events were the main factors found to be associated with a considerable impairment of caregivers' QOL in earlier studies (11, 20, 35, 36). Severe positive symptoms of schizophrenia such as hallucination, delusion, aggressiveness and destructive behavior are known to cause distress to the caregivers and lower their QOL (10, 12). It is suggested that the threatening nature of positive symptoms even though they are more episodic, as compared to the less threatening negative symptoms even though they are more chronic, make them more intolerable to caregivers (11, 12).

Wolthaus et al showed that disorganized symptoms in schizophrenia were predominant in causing caregiver burden. Furthermore, caregiver also need to cope with the unexpected symptoms of schizophrenia like delusions, hallucinations, cognitive defects, mood changes and also the social stigma related with the disease (14, 18). Stigma was not only been targeted on patients of schizophrenia, but was also referred upon persons close to the patients such as relatives, close friends and caregiver including mental health professionals (37).

The level of care burden generally reflects the QOL in caregivers (38, 39). Higher level of burden may come from longer duration of contact with the patient after they developed the illnesses (16)(40). This is reflected in this present study that later onset of illness and therefore shorter duration of caregiving is associated with better QOL. Patients with later onset of illness generally have milder symptoms as compared to those with earlier onset, therefore, explaining the better QOL of their caregivers (41).

The caregivers of patients who received more intensive care such as attending day care program reported significantly lower QOL. This probably due to more complex illness and higher needs in those patients as compared to their counterparts not needing an extra care, therefore, explaining the higher burden and lower QOL in their caregivers(36). However, this observation may be due lack of effectiveness of the current day care program. Therefore, further evaluation of the program in reducing the caregivers' burden would be very useful to answer this question. Other patient factors such as younger age, gender, financial aid and marital status were not significantly associated with QOL among caregivers of people with schizophrenia. Similar results were reported by past researchers (42).

Caregivers with certain characteristics are found to experience higher QOL in this study. Higher educational level appears to provide an advantage to the caregivers. It is speculated that better knowledge give rise to better capability to cope with their caregiving task as well as other stressors in life (10, 43). Caregivers with higher education possibly have more secured job and stable financial income that reduce the financial burden on caregiving task, thus improving their satisfaction for lives. Besides the extra income that work provides, it also serves as a respite or a diversion from caregiving task (ref).

Having medical problems was a disadvantage to the caregivers that affects their QOL in physical and social domains. This result is also similar to that found in earlier studies(10). This may be explained by the fact that physically unhealthy or sick caregivers would understandably less able to discharge their caregiving task and face more difficulties. In addition, this study found caregivers with higher stress level due to other concurrent psychosocial stressors had lower QOL. Apart from the illness, high pressure such as changing of jobs, lacking of social support and interpersonal conflicts were found to be associated with lower QOL (38).

As this study only include local population sample, the findings only represent the family caregiver population in the immediate locality. However it provides some useful information to the family caregivers and the service providers. Such information can assist the family caregivers to understand factors that might be contributing to their lower QOL and promotes their own prevention strategies. For service providers, supporting the caregivers in some of these modifiable factors in clinical practice is important to achieve their higher level QOL.

## Conclusion

At present, the family interventional program is already an important element of community-based mental health services in Malaysia. The current focus of the service to improve the overall wellbeing of patient and families with high risk factors especially those who are dealing with more severe illness, having medical problems and distress due to other concurrent life events should be continued. However, further local studies are needed to assess the effect of such interventional program on caregivers using either case-control or prospective approach and in a more representative sample of Malaysian families.

## Competing interests

The authors declare that they have no competing interests.

## Authors' contributions

RZ, MM, LSH and EJY were involved in writing of this article. LSH, EJY, SNAA, SFAA, MSB were involved in data collection. RSMR did the data analysis. The authors read and approved the final manuscript.
